# N-Doped Mesoporous Carbon Prepared from a Polybenzoxazine Precursor for High Performance Supercapacitors

**DOI:** 10.3390/polym13132048

**Published:** 2021-06-22

**Authors:** Periyasamy Thirukumaran, Raji Atchudan, Asrafali Shakila Parveen, Madhappan Santhamoorthy, Vanaraj Ramkumar, Seong-Cheol Kim

**Affiliations:** School of Chemical Engineering, Yeungnam University, Gyeongsan 38541, Korea; thiru.kumaran999@gmail.com (P.T.); atchudanr@gmail.com (R.A.); shakilaasraf@gmail.com (A.S.P.); santham83@gmail.com (M.S.); sriram27@gmail.com (V.R.)

**Keywords:** polybenzoxazine, calcinations, activation, nitrogen-doped porous carbons

## Abstract

Supercapacitors store energy either by ion adsorption or fast surface redox reactions. The capacitance produced by the former is known as electrochemical double layer capacitance and the latter is known as pseudo-capacitance. Carbon materials are found to be attractive materials for energy storage, due to their various micro-structures and wide source of availability. Polybenzoxazine (Pbz) is used as a source to produce carbon materials, due to the fact that the obtained carbon will be rich in N and O species for enhanced performance. Moreover, the carbon materials were produced via template-free method. In general, activation temperature plays a main role in altering the porosity of the carbon materials. The main purpose of this study is to find the suitable activation temperature necessary to produce porous carbons with enhanced performance. Considering these points, Pbz is used as a precursor to produce nitrogen-doped porous carbons (NRPCs) without using any template. Three different activation temperatures, namely 700, 800 and 900 °C, are chosen to prepare activated porous carbons; NRPC-700, NRPC-800 and NRPC-900. Hierarchical micro-/ meso-/macropores were developed in the porous carbons with respect to different activation temperatures. PBz source is used to produce carbons containing heteroatoms and an activation process is used to produce carbons with desirable pore structures. The surface morphology, pore structure and binding of heteroatoms to the carbon surface were analyzed in detail. NRPCs produced in this way can be used as supercapacitors. Further, electrodes were developed using these NRPCs and their electrochemical performance including capacitance, specific capacitance, galvanic charge/discharge, impedance, rate capability are analyzed. The obtained results showed that the activation temperature of 900 °C, is suitable to produce NRPC with a specific capacitance of 245 F g^−1^ at a current density of 0.5 A g^−1^, that are attributed to high surface area, suitable pore structure and presence of heteroatoms.

## 1. Introduction

Owing to environmental pollution concerns and the fossil fuel crisis, there is an increasing demand in the development of energy production and proper storage system [1,2,3]. Supercapacitors (SCs), also known as electrochemical capacitors (ECs), are energy storage devices that store electrical energy by accumulation of charges at the electrode/electrolyte interface [4,5,6]. Compared to conventional capacitors, supercapacitors have the capability to provide high specific power (10 kW/kg), long life cycle (>10^5^ cycles), fast charge/discharge process (a few seconds), environmental friendliness and relatively low price [5]. However, they do possess a lower energy density (5 Wh/kg) than Li-ion batteries (180 Wh/kg). Therefore, one of the most critical aspects is how to enhance their energy density while retaining their intrinsic specific power. Most promising applications of SCs include portable electronic devices, heavy construction-equipments, solar panels, memory back-up systems, emergency exits and so on [6,7,8].

Based on the energy storage mechanism, supercapacitors are classified into two categories, i.e., electrical double-layer capacitor (EDLC) and pseudo-capacitor. In EDLC, the capacitance arises from the electrostatic charge accumulated at the electrode/electrolyte, where carbon-based materials act as electrodes, whereas in a pseudo-capacitor, the capacitance arises from the Faradaic charge transfer reactions occurred due to electro-active species [9,10,11,12,13,14]. Recently, carbon-based materials have been used as electrode materials due to their desirable physical and chemical properties, including different existing forms, various micro-textures, large specific surface area, high electrical conductivity, excellent chemical stability, controllable porosity, easy process-ability and low cost [4,6]. Activation of carbon materials is an effective method to increase their surface area and control their pore size. Besides these, an appropriate pore structure also contributes for an ideal supercapacitor. In this regard, tailorable hierarchical structure containing micropores (<2 nm), mesopores (2–50 nm) and macropores (>50 nm) is much appreciated for better SC performance [3,5]. The electrochemical performance of carbon materials depends not only on their porosity, but also on the electrical conductivity and surface functionality. Therefore, the selection of carbon precursors containing heteroatoms, particularly oxygen and nitrogen functionalities, can enhance the capacitive performance by controlling the pseudo-capacitance effects. Porous carbon is obtained either from natural carbon precursors, such as coconut shell or synthetic carbon precursors [3,10,11] such as phenolic resins and organic polymers [15,16,17,18].

For the past few years, polymer-based porous carbons with nitrogen functionalities have been used as electrode materials for supercapacitor applications. The in-situ method of preparing porous carbon with N atom is the most preferred and costs much less [19]. Polybenzoxazines (Pbzs) are considered an advanced form of phenolic resins. Their striking features including self-polymerization, molecular design flexibility, and excellent mechanical and thermal stability gained interest for these kinds of materials. Enormous numbers of benzoxazine derivatives can be produced by varying the amine and phenolic precursors [20,21,22,23,24]. Moreover, oxygen and nitrogen functionalities can be easily incorporated into Pbz network structures, which in turn can act as a suitable carbon precursor [25,26]. Considering the properties of Pbzs, they can be used as a promising precursor to produce nitrogen rich porous carbons. In this work polybenzoxazines containing oxygen and nitrogen groups were synthesized through a Mannich reaction. Further, without using any template, the synthesized Pbz itself act as a carbon precursor to produce porous carbons by simple chemical activation method. The synthesis of porous carbon using template method is highly expensive, time-consuming and complicated, where the template itself has to be sacrificed. Hence, template-free synthesis is the best way to solve this issue [27,28,29,30,31]. The electrochemical properties were studied with respect to surface area, pore structure and pore volume and discussed in detail.

## 2. Synthesis of Benzoxazine Monomer and NRPCs

**Scheme 1:**
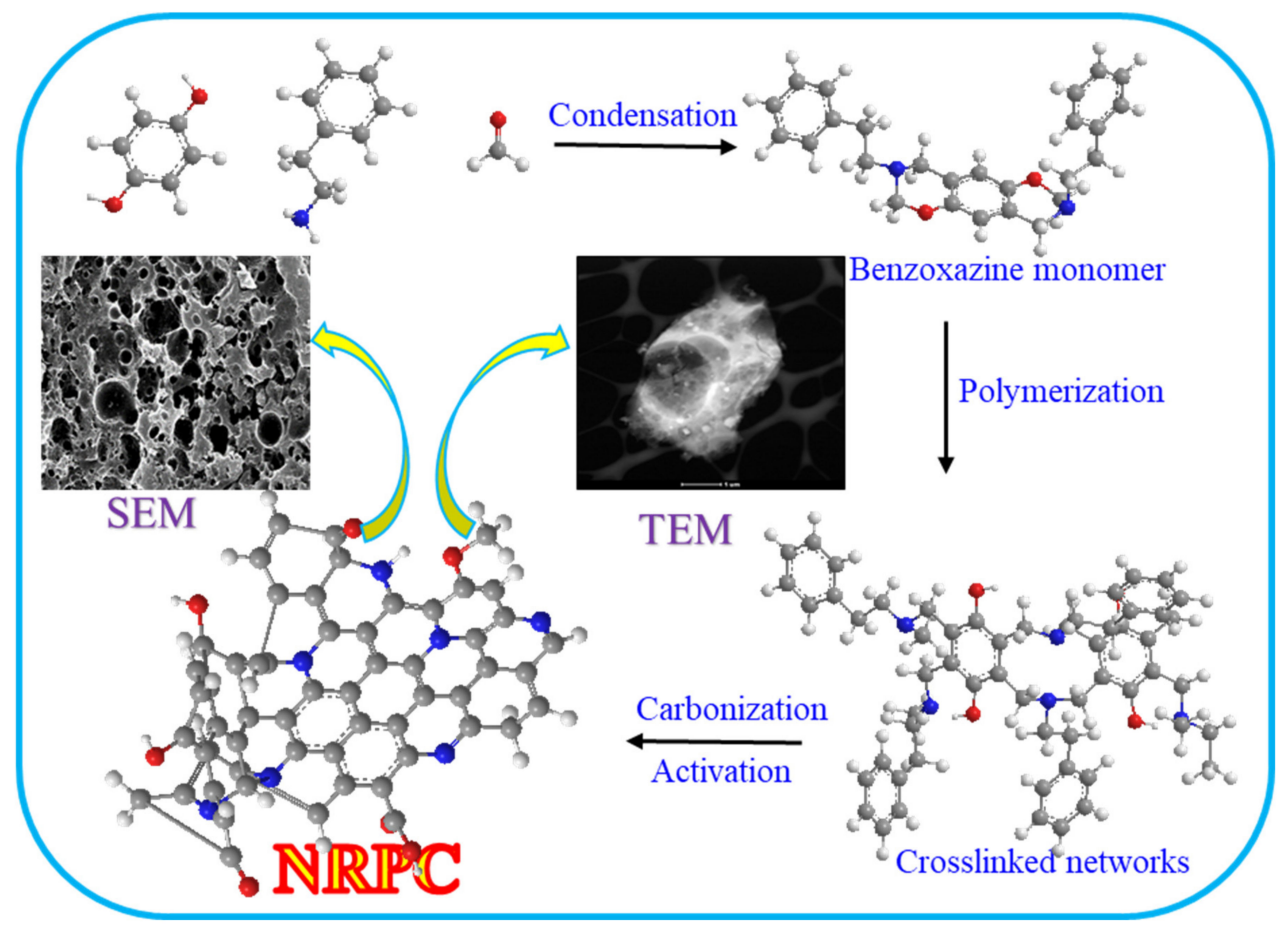
Synthesis of HPh-Bzo and preparation of NRPC.

The benzoxazine monomer (HPh-Bzo) was synthesized from hydroquinone, phenylethylamine and paraformaldehyde through the Mannich reaction according to a previously reported procedure [32] with some modifications. A detailed procedure is given in the Supporting Information (Scheme S1). For the preparation of nitrogen-doped porous carbons (NRPCs), the obtained HPh-Bzo monomer was subjected to curing/self-polymerization, carbonization and activation. The carbon materials obtained after these processes is free from impurities and results in the formation of well porous structure. Curing was done by stepwise heating of the HPh-Bzo monomer in an oven, following a temperature ramp of 100, 150, 180, 220 and 250 °C for 4 h at each temperature. Curing results in the formation of the polymer, i.e., polybenzoxazine. Carbonization was carried out by heating polybenzoxazine at 600 °C under an Ar atmosphere for 5 h at a heating rate of 1 °C/min. Further, the obtained carbonized material was soaked in an aq. KOH solution overnight (the weight of KOH used was twice the weight of the carbonized sample). It was then filtered and dried at 120 °C. Three different temperatures (say 700, 800 and 900 °C) were used to carry out the activation process. Carbonized samples were placed in a tube furnace and activation was done by heating at 700 °C in an Ar atmosphere with a ramp rate of 3 °C/min. In a similar way, activation of carbonized samples was also done at 800 and 900 °C. After activation, the products were repeatedly washed with 1 M HCl and deoinized H_2_O until a neutral pH was obtained [33,34,35,36,37]. They were then dried at 110 °C for 12 h. The dried samples were denoted as NRPC-700, NRPC-800 and NRPC-900 (Scheme 1). Yield of NRPC-700: 45%, NRPC-800: 42%, and NRPC-900: 40%

## 3. Results and Discussion

**Figure 1:**
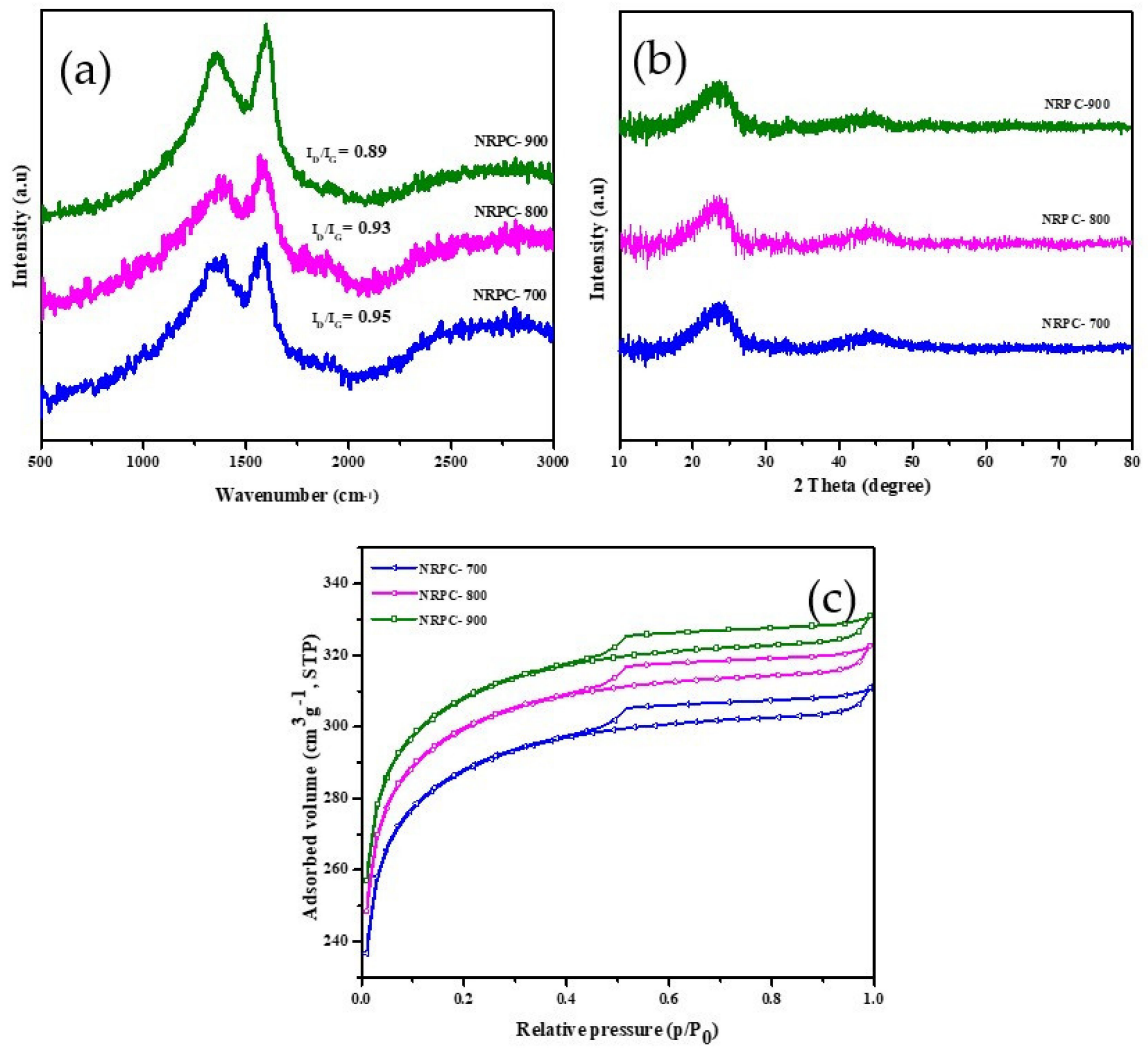
(**a**) Raman spectra, (**b**) XRD and (**c**) N2 adsorption isotherms of NRPCs.

The precursor of the polybenzoxazine-based nitrogen-doped carbons was synthesized by a Mannich reaction. Novel porous carbons with high surface area were obtained after carbonization and activation processes. Fourier transform infrared (FTIR), ^1^H-NMR and ^13^C-NMR spectra of the benzoxazine monomer (HPh-Bzo) were recorded and displayed in Figures S1–S3. The structural characteristics and graphitic property of the prepared activated carbon materials were determined by Raman and XRD analysis. Figure 1a represents the Raman spectra of NRPC-700, NRPC-800 and NRPC-900 carbons. As depicted in Figure 1a, all three samples show two strong peaks at 1356 and 1583 cm^−1^ corresponding to the ‘D’ band and ‘G’ band, respectively [12]. An intense ‘D’ band is observed for NRPC-700, which indicates that the NRPC-700 has disordered structure due to the presence of a greater number of heteroatoms. The degree of graphitization as represented by I_D_/I_G_ was found to be 0.95, 0.93 and 0.89 for NRPC-700, NRPC-800 and NRPC-900, respectively. This result reveals that the structural order and degree of graphitization increased with increasing the activation temperature from 700–900 °C [38]. Figure 1b depicts the XRD patterns of NRPC-700, NRPC-800 and NRPC-900, respectively. As shown in Figure 1b, two broad peaks at 2θ = 24.1 and 44.5° were typically observed for graphitic carbon materials, due to the diffraction of (002) and (100) planes, respectively. On the other hand, the NRPC-900 sample shows a gradually decreased peak intensity at 2θ = 24.1° (100), clearly indicating that there is an increased degree of graphitization. Further, Bragg’s equation was used to calculate the d-spacing value for graphitic carbon and is found to be 0.37 nm. The obtained d-spacing value is found to be higher than the conventional graphite and therefore, we assumed that these materials may contribute to enhanced capacity of supercapacitors when used as electrodes [8,12].

Nitrogen adsorption-desorption isotherms of the activated carbons are shown in Figure 1c. It could be seen that all the activated carbons—NRPC-700, NRPC-800 and NRPC-900—exhibit type IV isotherms with similar hysteresis loops in the mesopore range. A sharp rise in N_2_ isotherms were observed at a relative pressure below 0.1, representing micro-pore adsorption, which increases along with increased activation temperature. When compared with NRPC-700 and NRPC-800, the NRPC-900 sample showed a wider micropore size distribution which might be due to the higher activation temperature. A steady increase of adsorption isotherms were observed with increasing relative pressure up to P/P_o_ = 0.9, which might be due to the gradual adsorption of N_2_ molecules on the mesopores [25,39]. A sharp rise in the isotherms were observed above the relative pressure of 0.9 (P/P_0_), indicating the existence of macropores. From the BET isotherms, the specific surface areas of NRPC-700, NRPC-800 and NRPC-900 carbons are calculated to be 865.3, 980.6 and 1192.8 m^2^ g^−1^, respectively. The average mesopore size was slightly increased with increasing activation temperature from 700 to 900 °C, indicating that the mesopores were enlarged by KOH etching under severe activation conditions. In addition, macropores with pore sizes ranging from 50 to 100 nm for were generated for NRPC-700, NRPC-800 and NRPC-900 by KOH corrosion and nitrogen and oxygen heteroatom functionalities were retained even at this high temperature. As polybenzoxazines possess high chemical and thermal stability, and exhibit high char yield (around 50%), all the samples NRPC-700, NRPC-800 and NRPC-900 prepared using PBz were also obtained at high yield.

**Figure 2:**
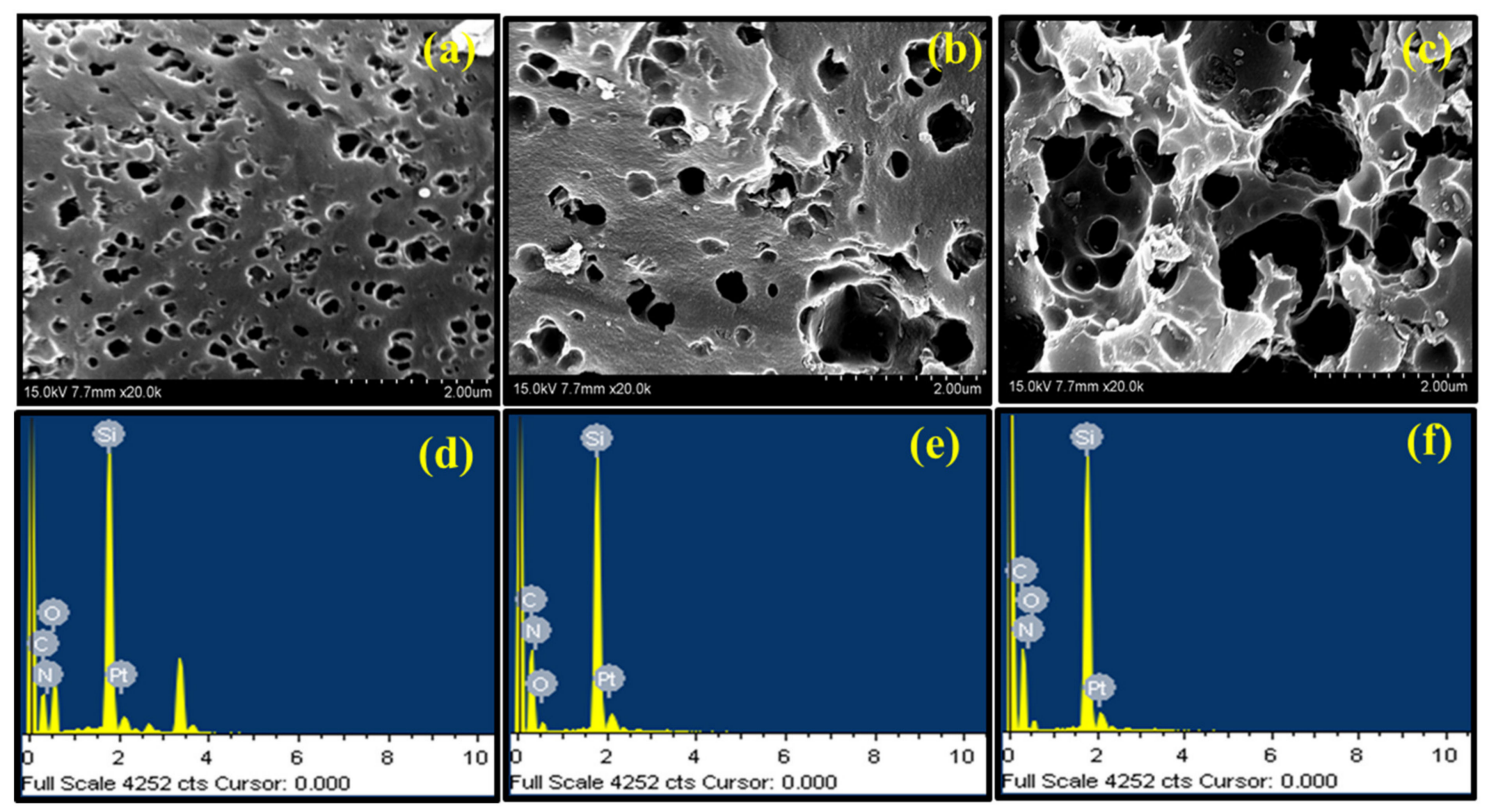
SEM images and EDX of (**a**,**d**) NRPC-700, (**b**,**e**) NRPC-800 and (**c**,**f**) NRPC-900.

The surface morphologies of the activated carbons at different activation temperatures are shown in Figure 2. As observed from the SEM images (Figure 2a–c), the thermal-induced activation process results in the formation of carbon materials with rough surfaces and varying pore sizes [25]. For instance, the porous carbon sample NRPC-700 obtained at a low activation temperature of 700 °C, consists of many irregular micropores with various sizes, and it also displays the existence of very few mesopores (Figure 2a). In contrast, at an increased activation temperature (say 800 °C), NRPC-800 develops a considerably more rough surface with an increased number of mesopores and with very few macropores in the sample (Figure 2b).

**Figure 3:**
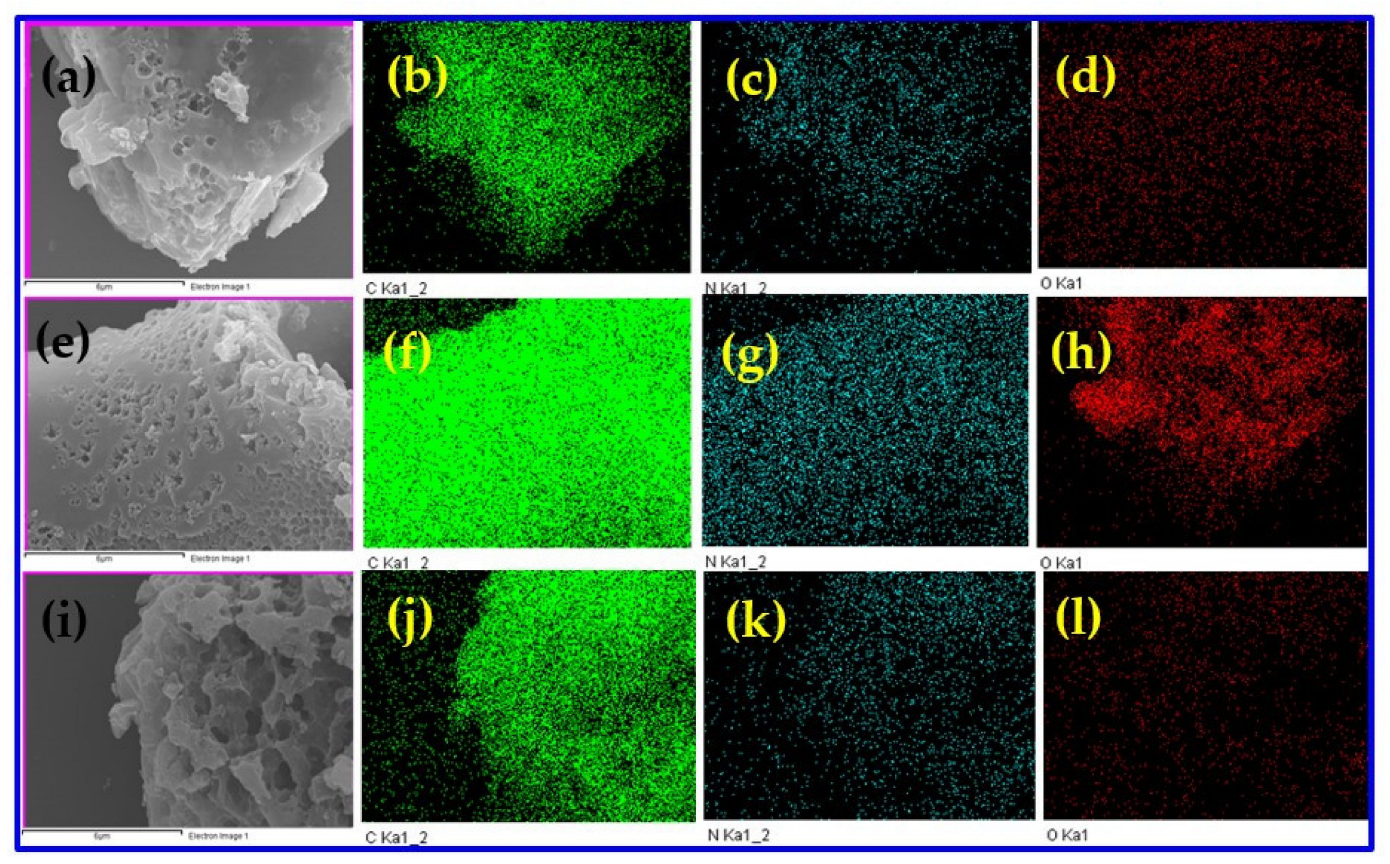
FESEM images with corresponding elemental mapping of (**a**–**d**) NRPC-700, (**e**–**h**) NRPC-800 and (**i**–**l**) NRPC-900.

**Figure 4:**
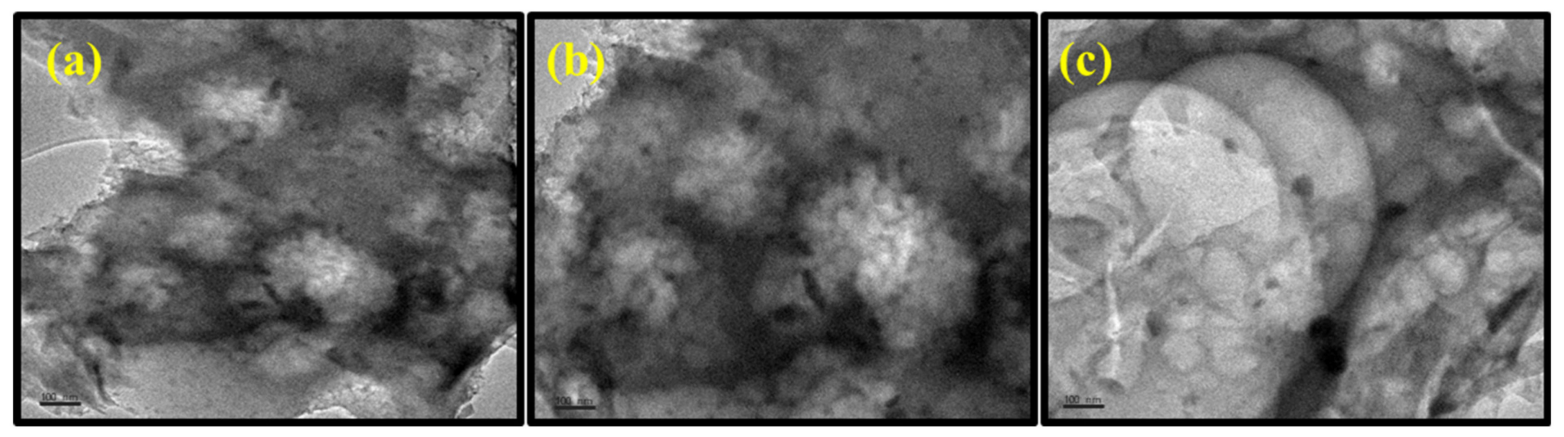
TEM images of (**a**) NRPC-700, (**b**) NRPC-800 and (**c**) NRPC-900.

Furthermore, at a still higher activation temperature (say 900 °C), NRPC-900 has a collapsed structure with many voids and macropores with pore sizes ranging between 0.5 to 1.0 µm (Figure 2c). Many macropores were developed on the surface of the carbon, which in-turn leads to thinner pore walls [2,40,41,42,43]. These results evidenced that the pore structure and pore size distribution can be tailored by optimizing the activation temperature. The elemental compositions of the NRPC-700, NRPC-800 and NRPC-900 were confirmed by energy-dispersive X-ray spectroscopy (EDX) analysis (Figure 2d–f). FESEM images and the corresponding elemental mapping are shown in Figure 3a–l, indicating that the carbon surface has a network morphology and is composed of compact particles containing the elements carbon, oxygen and nitrogen. Also, the elemental mapping results evidence that there is a uniform distribution of nitrogen within the carbon matrix. These results further confirm that the synthesized carbon material is doped with nitrogen. The morphology of NRPC-700, NRPC-800 and NRPC-900 samples was further confirmed by TEM analysis. Figure 4a–c show the TEM images of the synthesized NRPC-700, NRPC-800 and NRPC-900 materials with open-pore networks that are desirable for supercapacitor applications. These kinds of nanoarchitectures not only shorten diffusion pathways to accelerate ion transport, but also provide a continuous electron pathway for electrical contact. Moreover, the samples NRPC-800 and NRPC-900 showed an increased number of pores with regular arrangements, indicating the existence of sp^2^-bonded carbon that especially favours the electrical conductivity of carbon materials. Therefore, the existence of hierarchical porous structures that were observed from TEM images is consistent with the SEM observations.

**Figure 5:**
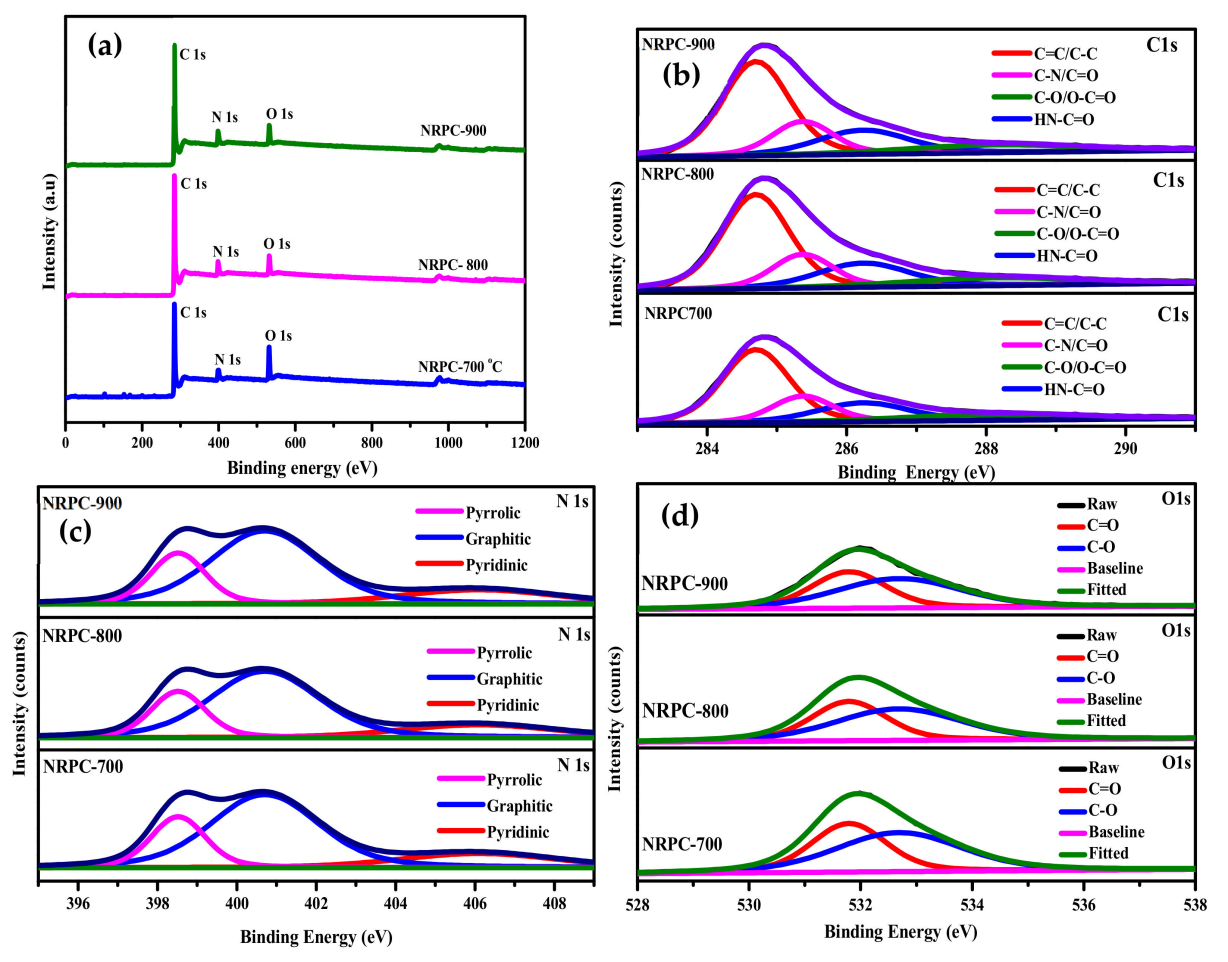
(**a**) XPS spectra and (**b**–**d**) C1s, N1s and O1s spectra of NRPCs.

The activated porous carbon materials derived from polybenzoxazines contain relatively higher content of nitrogen species about 3.64–6.26 wt.% and oxygen species about 10.61–13.65 wt.%. The presence of these heteroatoms (N and O atoms) effectively improves the wettability and electrical conductivity of the prepared carbon materials and thus facilitates the accessibility of the electrolyte ions, thereby enhancing their capacitance performance. The XPS analysis data (Figure 5) representing the chemical state of carbon, nitrogen and oxygen species that exist in the porous carbon materials. As seen in Figure 5, the XPS curves exhibited by the NRPC-700, NRPC-800 and NRPC-900 samples show three peaks corresponding to C 1s, N1s and O1s, respectively. Therefore, the XPS result implies a strong evidence for the existence of nitrogen and oxygen atoms in the prepared carbon materials, which is expected to be derived from the benzoxazines monomers when employing them as the carbon source. These results indicate that there was no impurity in the synthesized nitrogen rich porous carbon.

Figure 5a shows the XPS survey scans of the NRPC-700, NRPC-800 and NRPC-900 materials. The spectra contain C, N and O photoelectron peaks with binding energies of approximately 285.7, 399.2 and 531.8 eV, respectively. Further, analyses were performed on C 1s, N 1s and O 1s signals and are shown in Figure 5b–d. The fitting of the C 1s spectrum (Figure 5b) was resolved into four peaks, the first peak appeared at 284.7 eV is assigned to the hydrocarbon chains of (C=C/C–C), the second peak appeared at 285.4 eV is due to carbon atoms in the C-N bond, the third peak appeared at 286.3 eV is due to the carbon atom bonded with O and N (HN–C=O) groups and the fourth peak appeared at 287.5 eV, corresponding to the characteristic peak of O–C=O/C=N/C–OH groups.

Figure 5c represents the presence of different nitrogen species bound to the activated porous carbons. Three different nitrogen species are observed, at 398.4 eV due to pyrrolic or pyridinic nitrogen; at 400.5 eV due to quaternary nitrogen; and at 405.1 eV due to oxidized nitrogen. The activated porous carbons contain a higher amount of pyridinic and pyrrolic nitrogen species, which could possibly result from the polybenzoxazine source used. Moreover, these pyridinic and pyrrolic nitrogen species becomes electrochemically active in an acidic aqueous solution and thus provide an increased capacitance. On the other hand, the quaternary nitrogen species present in the interior of the carbon matrix effectively promote electron transfer and therefore, improve the conductivity. In addition to this, the improved conductivity of NRPC samples also depends on their degree of graphitization. Therefore, among the three activated carbons, the sample NRPC-900 (activated at a higher temperature of 900 °C), containing more thermally stable quaternary nitrogen groups and possessing a higher degree of graphitization which further enhanced the electrical conductivity. The O1s spectrum (Figure 5d) deconvoluted into two binding energy peaks appearing at 531.4 and 533.0 eV revealing the presence of quinone (Ph=O), phenolic and hydroxyl or ether (C–O–C), chemisorbed oxygen or water functional groups, respectively. Among these oxygen functional groups, quinone groups in the carbon matrix are not electrochemically active in the reversible redox reactions in an alkaline medium. However, phenolic hydroxyls (by reduction) and hydroxyls or ethers (by deprotonation) exhibit quasi-reversible pseudocapacitances [44,45,46,47]. Hence, the prepared NRPCs with high contents of phenolic hydroxyls or ether oxygens and carbonyl oxygens can generate high pseudo-capacitance in an acidic alkaline electrolyte.

## 4. Electrochemical Measurements

**Figure 6:**
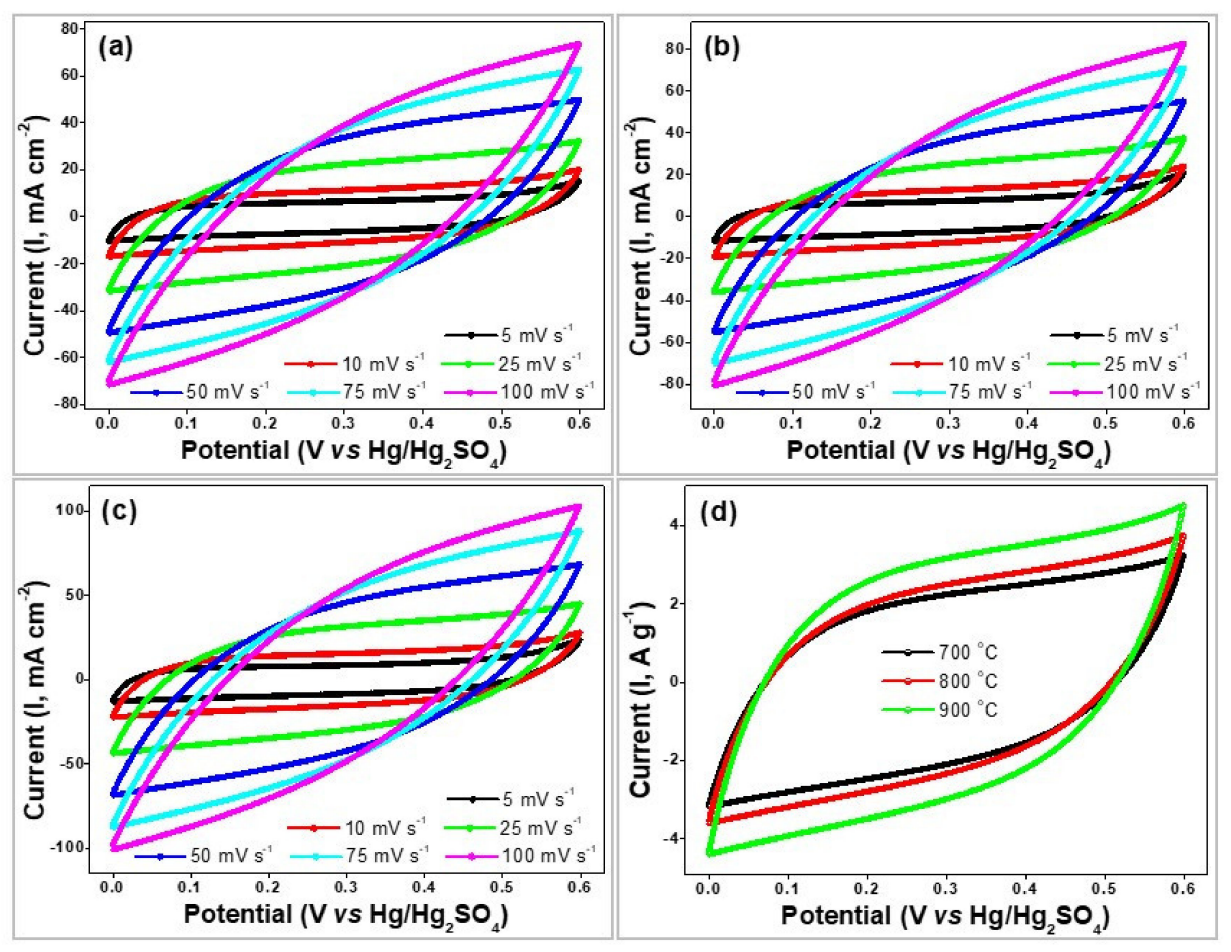
CV curves of NRPCs with different scan rates (**a**) NRPC-700, (**b**) NRPC-800, (**c**) NRPC-900 and (**d**) CV curves of NRPCs at a scan rate of 25 mV s^−1^.

All the abovementioned results show that electrode materials can be prepared from the NRPC materials. To verify this, the prepared carbon materials were examined by a three-electrode system using 1 M H_2_SO_4_ aqueous electrolyte and their electrochemical performance were analyzed. Figure 6a–c show the cyclic voltammetry (CV) curves of the NRPC-700, NRPC-800 and NRPC-900 materials, respectively, in 1 M H_2_SO_4_ aqueous electrolyte with a working voltage range of 0.0–0.6V and Figure 6d shows the CV curves of all the three electrodes at a scan rate of 25 mV s^−1^. The CV curves of all the three electrodes showed a quasi-rectangular profile within 0.0–0.6 V at a scanning rate ranging from 5 to 100 mV s^−1^, and almost near-rectangular shape is well maintained at a scan rate of 25 mV s^−1^. Observed results evidenced that the prepared materials have perfect capacitive behaviour. At a low scan rate, all of the NRPC electrodes showed a quasi-rectangular shape, exhibiting a small electrical double-layer contribution (EDLC) due to lower specific surface. On the other hand, a near-rectangular shape was observed for all the electrodes at a scan rate of 25 mV s^−1^, indicating the existence of pseudo-capacitive reactions, due to higher nitrogen contents. However, at higher scan rate (say 100 mV s^−1^), all the NRPC electrodes still maintain a less rectangular shape, implying a good capacitive performance for charge/discharge operations. As compared to the sample NRPC-900, CV curves of NRPC-700 and NRPC-800 electrodes become highly distorted at higher scan rates. This could be due to the presence of micropores which restrict fast ionic transportation through them. In contrast, the NRPC-900 electrode containing enormous macropores and few mesopores favours the quick electrolyte diffusion into their interior surface and provides an improved capacitance performance [6,8,26].

**Figure 7:**
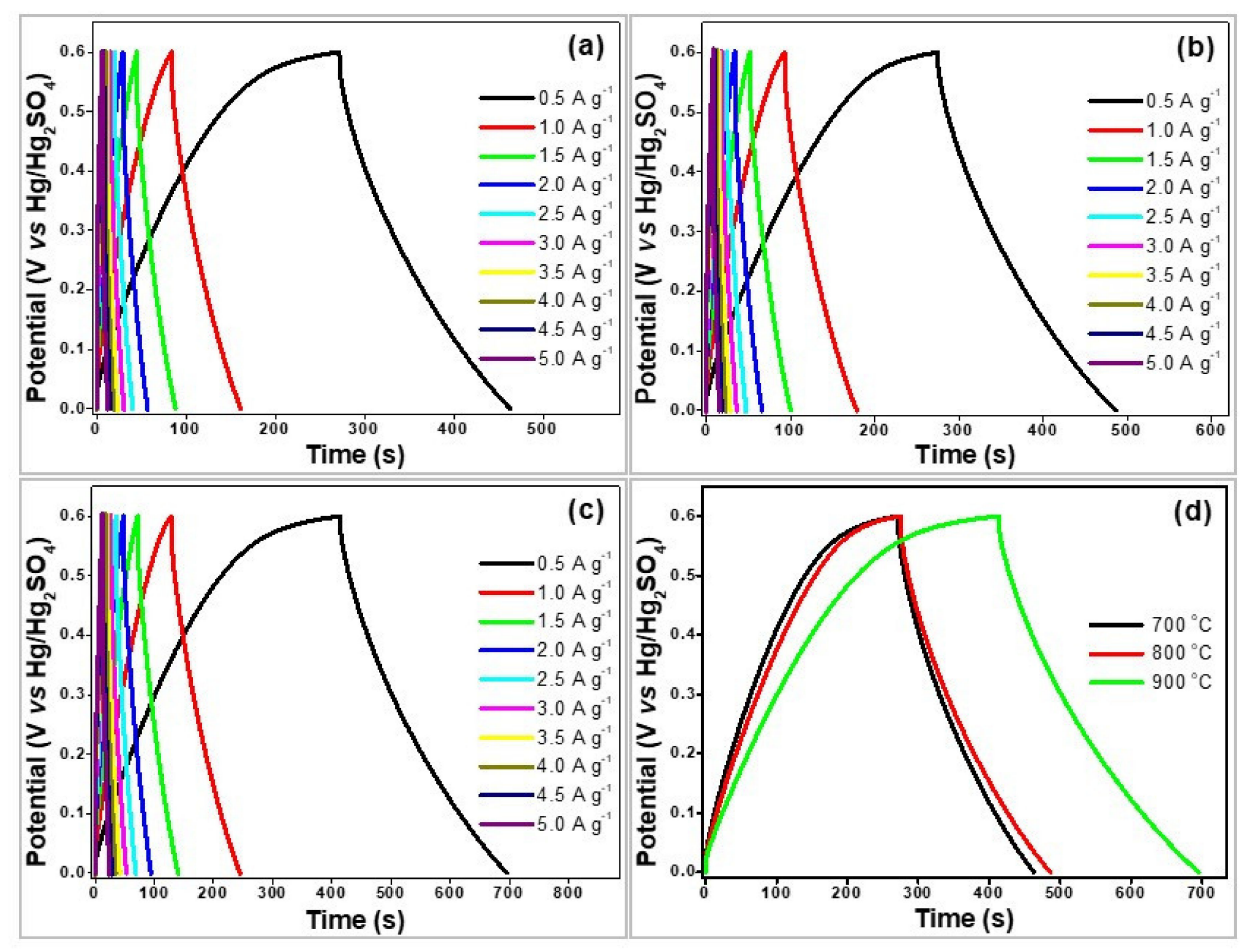
GCD curves of NRPCs at different current densities (**a**) NRPC-700, (**b**) NRPC-800, (**c**) NRPC-900 and (**d**) GCD curves of NRPCs at a current density of 0.5 A g^−1^.

Figure 7a–c display the GCD curves with a linear shape possessing small curvature for all the NRPC electrodes, indicating good capacitive properties and electrochemical reversibility. The NRPC-900 electrode displays a considerably longer discharge time when compared with NRPC-700 and NRPC-800. This might be due to the presence of macropores and mesopores, which not only make the inner surface area more electrochemically accessible for electrolyte ions, but also facilitate a fast diffusion of electrolyte ions in the pore channels at high current densities. The higher activation temperature of 900 °C for NRPC-900 results in producing highly porous structure with sufficient amount of electrochemically active nitrogen and oxygen atoms in it, which leads to increased pseudo-capacitance [6,48,49,50,51,52]. The electrochemical performance obtained from GCD and CV measurements are in good agreement with each other. Figure 7d shows the GCD of all the three electrodes at 0.5 A g^−1^. The curve indicates that the NRPC-900 electrode shows a high discharge time when compared with NRPC-800 and NRPC-700 samples because of their wider micropore distribution and the presence of meso/macropores. All the obtained results confirmed that the pronounced contribution of pseudo-capacitance for NRPC-700, NRPC-800 and NRPC-900 electrodes.

**Figure 8:**
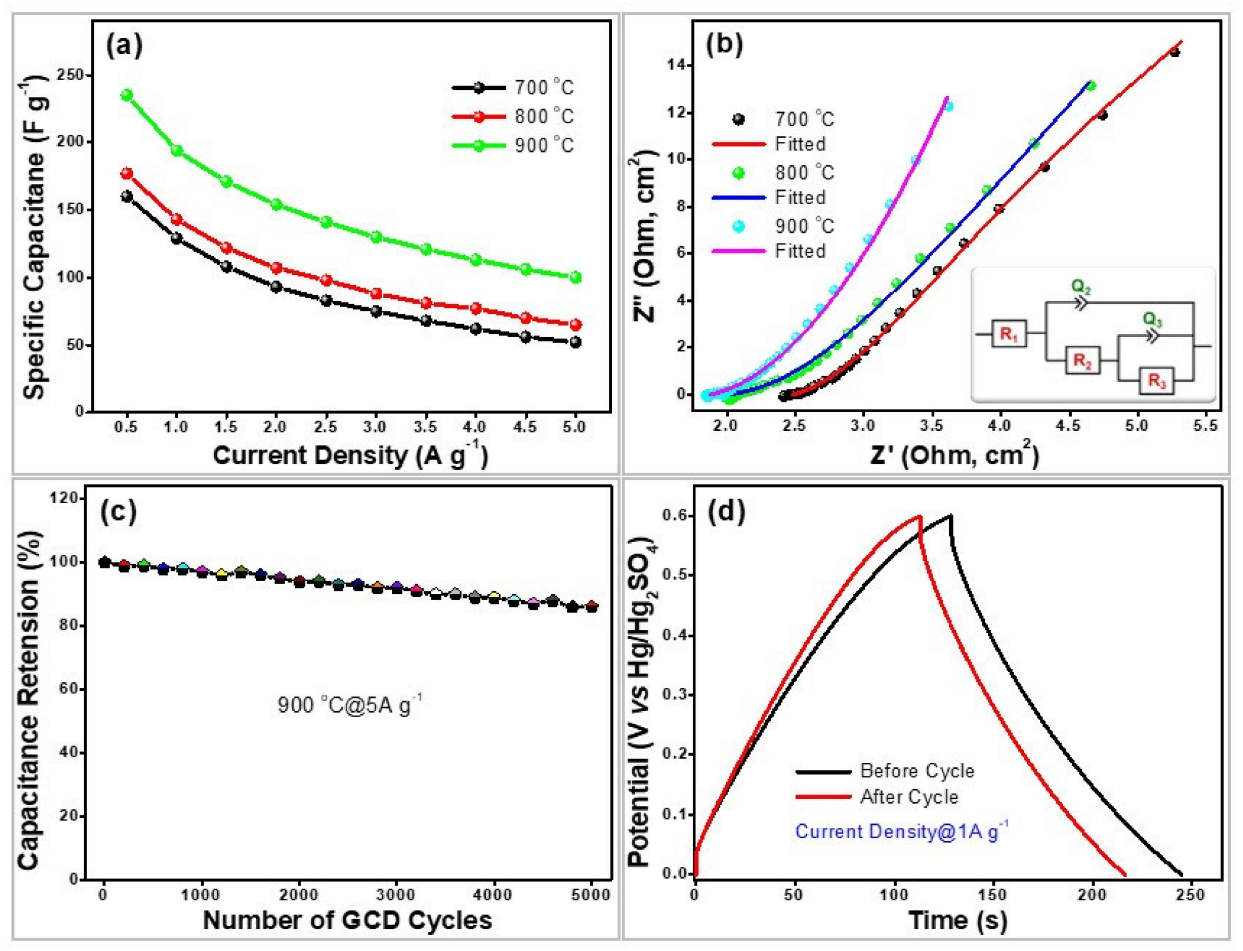
(**a**) Specific capacitance of NRPCs, (**b**) EIS showing Nyquist plots of NRPCs (**c**) Long-term cycling stability of NRPC-900 at a current density of 5 A g^−1^ and (**d**) GCD curves of NRPC-900 before and after 5000 cycles at a current density of 1 A g^−1^.

Figure 8a displays the specific capacitance of the NRPC-700, NRPC-800 and NRPC-900 electrodes, respectively, at different current densities. It could be seen that with a current density from 0.5 to 5.0 A g^−1^, the specific capacitance of the NRPC-700, NRPC-800 and NRPC-900 electrodes were found to be 160–50 F g^−1^, 175–70 F g^−1^ and 245–125 F g^−1^, respectively. Among, the sample NRPC-900 exhibits highest specific capacitance owing to their porous structure [8]. The presence of micropores, less developed surface area, low porosity and large diffusion resistance results in poor capacitance of NRPC-700 and NRPC-800 electrodes. Even though all the electrodes contain heteroatoms such as nitrogen and oxygen, the presence of meso-/macropores and the accessibility of the carbon surface for diffusion of the electrolyte ions play an important role in improving their pseudo-capacitance [53,54,55,56]. In this view, NRPC-900 satisfies all the necessary requirements and hence shows improved capacitance. The Nyquist plots of all the three electrodes are shown in Figure 8b. Similar shaped Nyquist plots were observed for all the three electrodes. At high frequency region, a depressed semicircle and at low frequency region, a gradual slope was found indicating good capacitive behaviour. When compared with NRPC-700 and NRPC-800 samples, NRPC-900 sample show a short and less gradual slope implying lower diffusion resistance [57,58,59]. Moreover, the charge transfer resistances of the NRPC-700, NRPC-800 and NRPC-900 electrodes were found to be 2.4, 2.0 and 1.5 Ω, respectively. The presence of meso-/macropores and high graphitization degree makes NRPC-900 electrodes possess high ionic conductivity than the other two electrodes.

Further, to evaluate the cycling stability of the NRPC-900 electrode, GCD studies were performed at a current density of 1 A g^−1^ in a 1 M H_2_SO_4_ aqueous electrolyte using a three-electrode system. The specific capacitance of the NRPC-900 electrode decreased from 194 to 172 F g^−1^ after 5000 cycles. It also showed a capacitance retention ratio of 89% (Figure 8c), indicating excellent long-term cycling durability. The cycling durability of the NRPC-900 electrode can be confirmed by the integral areas surrounded by the GCD curves of the 1st and 5000th cycles at 1 A g^−1^, as depicted in Figure 8d.

## 5. Conclusions

A simple Mannich condensation, self-polymerization, calcination and activation methods were used for the successful synthesis of nitrogen-doped porous carbons from polybenzoxazine. The change in activation temperature alters the morphology and surface chemistry of NRPCs, thereby influencing their supercapacitor performance. High degree of graphitization, hierarchical meso-/macropores and porous structure were developed at an activation temperature of 900 °C for the NRPC-900. The prepared porous carbons produced contain a high amount of nitrogen (6.26 wt%) and oxygen species (13.65 wt%) as evidenced by EDX and XPS analyses. Such high contents can only be produced through the polybenzoxazine source. Among the three prepared electrodes, NRPC-900 electrode exhibits superior electrochemical performance with a specific capacitance of 245 F g^−1^ at a current density of 0.5 A g^−1^ with 89% capacitance retention. These are attributed to high surface area, suitable porous structure, presence of nitrogen and oxygen functionalities and high graphitization degree. Moreover, good cycling durability is retained for over 5000 charge/discharge cycles. All the above results confirm that the activation temperature plays a prominent role in tailoring the porous structure of the carbon skeleton and therefore improves the supercapacitor ability by accumulation and diffusion of electrolyte ions. From all the experimental results obtained from this study, we believe that the prepared NRPC materials could be utilized as a promising electrode material for supercapacitor applications.

## Data Availability

The data presented in this study are available on request from the corresponding author.

## Supplementary Materials

The following are available online at https://www.mdpi.com/article/10.3390/polym13132048/s1, Scheme S1: Synthesized of benzoxazines monomers (HPh-Bzo), Figure S1: FT-IR spectrum benzoxazines monomers (HPh-Bzo), Figure S2: 1H-NMR spectrum benzoxazines monomers (HPh-Bzo), Figure S3: 13C-NMR spectrum benzoxazines monomers (HPh-Bzo).

## References

[1] Yuan C.Z., Gao B., Shen L., Yang S.D., Hao L., Lu X.J., Zhang F., Zhang L.J., Zhang X.G. (2010). Hierarchically structured carbon-based composites: Design, synthesis and their application in electrochemical capacitors. Nanoscale.

[2] Hong X., Hui K.S., Zeng Z., Zhang L., Mo M., Li M. (2014). Hierarchical nitrogen-doped porous carbon with high surface area derived from endothelium corneum gigeriae galli for high-performance supercapacitor. Electrochim. Acta.

[3] Zhai Y., Dou Y., Zhao D., Fulvio P.F., Mayes R., Dai S. (2011). Carbon Materials for Chemical Capacitive Energy Storage. Adv. Mater..

[4] Zhang L., Zhao X.S. (2009). Carbon-based materials as supercapacitor electrodes. Chem. Soc. Rev..

[5] Simon P., Gogotsi Y. (2008). Materials for electrochemical capacitors. Nat. Mater..

[6] Fic K., Meller M., Frackowiak E. (2014). Strategies for enhancing the performance of carbon/carbon supercapacitors in aqueous electrolytes. Electrochim. Acta.

[7] Inagaki M., Konno H., Tanaike O. (2010). Carbon materials for electrochemical capacitors. J. Power Sources.

[8] Xia K., Gao Q., Jiang J., Hu J. (2008). Hierarchical porous carbons with controlled micropores and mesopores for supercapacitor electrode materials. Carbon.

[9] Sawaryn C., Landfester K., Taden A. (2010). Benzoxazine Miniemulsions Stabilized with Polymerizable Nonionic Benzoxazine Surfactants. Macromolecules.

[10] Frackowiak E., Beguin F. (2001). Carbon materials for the electrochemical storage of energy in capacitors. Carbon.

[11] Huang J., Sumpter B., Meunier V. (2008). Theoretical Model for Nanoporous Carbon Supercapacitors. Angew. Chem. Int. Ed..

[12] Ania C., Khomenko V., Raymundo-Piñero E., Parra J.B., Beguin F. (2007). The Large Electrochemical Capacitance of Microporous Doped Carbon Obtained by Using a Zeolite Template. Adv. Funct. Mater..

[13] Hall P.J., Mirzaeian M., Fletcher S.I., Sillars F.B., Rennie A., Shitta-Bey G.O., Wilson G., Cruden A., Carter R. (2010). Energy storage in electrochemical capacitors: Designing functional materials to improve performance. Energy Environ. Sci..

[14] Hulicova-Jurcakova D., Kodama M., Shiraishi S., Hatori H., Zhu Z., Lu G.Q. (2009). Nitrogen-Enriched Nonporous Carbon Electrodes with Extraordinary Supercapacitance. Adv. Funct. Mater..

[15] Chmiola J., Yushin G., Dash R., Gogotsi Y. (2006). Effect of pore size and surface area of carbide derived carbons on specific capacitance. J. Power Sources.

[16] Liu X., Gu Y. (2002). Study on the volumetric expansion of benzoxazine curing with different catalysts. J. Appl. Polym. Sci..

[17] Agag T., Arza C.R., Maurer F.H.J., Ishida H. (2010). Primary Amine-Functional Benzoxazine Monomers and Their Use for Amide-Containing Monomeric Benzoxazines. Macromolecules.

[18] Sawaryn C., Landfester K., Taden A. (2011). Benzoxazine Miniemulsions Stabilized with Multifunctional Main-chain Benzoxazine Protective Colloids. Macromolecules.

[19] Thubsuang U., Chotirut S., Thongnok A., Promraksa A., Nisoa M., Manmuanpom N., Wongkasemjit S., Chaisuwan T. (2020). Facile preparation of polybenzoxazine-based carbon microspheres with nitrogen functionalities: Effects of mixed solvents on pore structure and supercapacitive performance. Front. Chem. Sci. Eng..

[20] Yagci Y., Kiskan B., Ghosh N.N. (2009). Recent advancement on polybenzoxazine-A newly developed high performance thermoset. J. Polym. Sci. Part. A Polym. Chem..

[21] Wan L., Wang J., Xie L., Sun Y., Li K. (2014). Nitrogen-Enriched Hierarchically Porous Carbons Prepared from Polybenzoxazine for High-Performance Supercapacitors. ACS Appl. Mater. Interfaces.

[22] Winter M., Brodd R.J. (2004). What Are Batteries, Fuel Cells, and Supercapacitors?. Chem. Rev..

[23] Nishihara H., Kyotani T. (2012). Templated Nanocarbons for Energy Storage. Adv. Mater..

[24] Xu B., Hou S., Cao G., Wu F., Yang Y. (2012). Sustainable nitrogen-doped porous carbon with high surface areas prepared from gelatin for supercapacitors. J. Mater. Chem..

[25] Li L., Liu E., Li J., Yang Y., Shen H., Huang Z., Xiang X., Li W. (2010). A doped activated carbon prepared from polyaniline for high performance supercapacitors. J. Power Sources.

[26] Kim C., Ngoc B.T.N., Yang K.S., Kojima M., Kim Y.A., Endo M., Yang S.C. (2007). Self-Sustained Thin Webs Consisting of Porous Carbon Nanofibers for Supercapacitors via the Electrospinning of Polyacrylonitrile Solutions Containing Zinc Chloride. Adv. Mater..

[27] Dutta S., Bhaumik A., Wu K.C.-W. (2014). Hierarchically porous carbon derived from polymers and biomass: Effect of interconnected pores on energy applications. Energy Environ. Sci..

[28] Deng Y., Liu C., Yu T., Liu F., Zhang F., Wan Y., Zhang L., Wang C., Tu B., Webley P.A. (2007). Facile Synthesis of Hierarchically Porous Carbons from Dual Colloidal Crystal/Block Copolymer Template Approach. Chem. Mater..

[29] Wang Z., Stein A. (2008). Morphology Control of Carbon, Silica, and Carbon/Silica Nanocomposites: From 3D Ordered Macro-/Mesoporous Monoliths to Shaped Mesoporous Particles. Chem. Mater..

[30] Han S., Hyeon T. (1999). Simple silica-particle template synthesis of mesoporous carbons. Chem. Commun..

[31] Wang S., Han C., Wang J., Deng J., Zhu M., Yao J., Li H., Wang Y. (2014). Controlled Synthesis of Ordered Mesoporous Carbohydrate-Derived Carbons with Flower-like Structure and N-Doping by Self-Transformation. Chem. Mater..

[32] Thirukumaran P., Shakila A., Muthusamy S. (2014). Synthesis and characterization of novel bio-based benzoxazines from eugenol. RSC Adv..

[33] Periyasamy T., Asrafali S.P., Muthusamy S. (2014). New benzoxazines containing polyhedral oligomeric silsesquioxane from eugenol, guaiacol and vanillin. New J. Chem..

[34] Thirukumaran P., Parveen A.S., Sarojadevi M. (2014). Synthesis and Copolymerization of Fully Biobased Benzoxazines from Renewable Resources. ACS Sustain. Chem. Eng..

[35] Thirukumaran P., Manoharan R.K., Parveen A.S., Atchudan R., Kim S.-C. (2019). Sustainability and antimicrobial assessments of apigenin based polybenzoxazine film. Polymers.

[36] Thirukumaran P., Atchudan R., Balasubramanian R., Parveen A.S., Kim S.-C. (2018). Direct synthesis of nitrogen-rich carbon sheets via polybenzoxazine as highly active electrocatalyst for water splitting. Int. J. Hydrogen Energy.

[37] Thirukumaran P., Atchudan R., Parveen A.S., Lee Y.R., Kim S.-C. (2018). Polybenzoxazine originated N-doped mesoporous carbon ropes as an electrode material for high-performance supercapacitors. J. Alloy. Compd..

[38] Zhang M., Chen M., Reddeppa N., Xu D., Jing Q.-S., Zha R. (2018). Nitrogen self-doped carbon aerogels derived from trifunctional benzoxazine monomers as ultralight supercapacitor electrodes. Nanoscale.

[39] Jin H., Wang X., Gu Z., Polin J. (2013). Carbon materials from high ash biochar for supercapacitor and improvement of capacitance with HNO3 surface oxidation. J. Power Sources.

[40] Yuan D., Chen J., Zeng J., Tan S. (2008). Preparation of monodisperse carbon nanospheres for electrochemical capacitors. Electrochem. Commun..

[41] Wang Q., Yan J., Wang Y., Ning G., Fan Z., Wei T., Cheng J., Zhang M., Jing X. (2013). Template synthesis of hollow carbon spheres anchored on carbon nanotubes for high rate performance supercapacitors. Carbon.

[42] Zhao Q., Wang X., Wu C., Liu J., Wang H., Gao J., Zhang Y., Shu H. (2014). Supercapacitive performance of hierarchical porous carbon microspheres prepared by simple one-pot method. J. Power Sources.

[43] Xu X., Zhou J., Nagaraju D.H., Jiang L., Marinov V.R., Lubineau G., Alshareef H.N., Oh M. (2015). Flexible, Highly Graphitized Carbon Aerogels Based on Bacterial Cellulose/Lignin: Catalyst-Free Synthesis and its Application in Energy Storage Devices. Adv. Funct. Mater..

[44] Wu J., Zhang D., Wang Y., Hou B. (2013). Electrocatalytic activity of nitrogen-doped graphene synthesized via a one-pot hydrothermal process towards oxygen reduction reaction. J. Power Sources.

[45] Andreas H.A., Conway B.E. (2006). Examination of the double-layer capacitance of an high specific-area C-cloth electrode as titrated from acidic to alkaline pHs. Electrochim. Acta.

[46] Fujisawa K., Cruz-Silva R., Yang K.-S., Kim Y.A., Hayashi T., Endo M., Terrones M., Dresselhaus M.S. (2014). Importance of open, heteroatom-decorated edges in chemically doped-graphene for supercapacitor applications. J. Mater. Chem. A.

[47] Kerisit S., Schwenzer B., Vijayakumar M. (2014). Effects of Oxygen-Containing Functional Groups on Supercapacitor Performance. J. Phys. Chem. Lett..

[48] Atchudan R., Edison T.N.J.I., Perumal S., Lee Y.R. (2017). Green synthesis of nitrogen-doped graphitic carbon sheets with use of Prunus persica for supercapacitor applications. Appl. Surf. Sci..

[49] Candelaria S.L., Garcia B.B., Liu D., Cao G., Candelaria S.L., Garcia B.B., Liu D., Cao G. (2012). Nitrogen modification of highly porous carbon for improved supercapacitor performance. J. Mater. Chem..

[50] Wang D.-W., Li F., Chen Z.-G., Lu G.Q., Cheng H.-M. (2008). Synthesis and Electrochemical Property of Boron-Doped Mesoporous Carbon in Supercapacitor. Chem. Mater..

[51] Guo Y., Shi Z.-Q., Chen M.-M., Wang C.-Y. (2014). Hierarchical porous carbon derived from sulfonated pitch for electrical double layer capacitors. J. Power Sources.

[52] Atchudan R., Perumal S., Karthikeyan D., Pandurangan A., Lee Y.R. (2015). Synthesis and characterization of graphitic mesoporous carbon using metal–metal oxide by chemical vapor deposition method. Microporous Mesoporous Mater..

[53] Lee Y.-H., Chang K.-H., Hu C.-C. (2013). Differentiate the pseudocapacitance and double-layer capacitance contributions for nitrogen-doped reduced graphene oxide in acidic and alkaline electrolytes. J. Power Sources.

[54] Yang X., Wu D., Chen X., Fu R. (2010). Nitrogen-Enriched Nanocarbons with a 3-D Continuous Mesopore Structure from Polyacrylonitrile for Supercapacitor Application. J. Phys. Chem. C.

[55] Wickramaratne N.P., Xu J., Wang M., Zhu L., Dai L., Jaroniec M. (2014). Nitrogen Enriched Porous Carbon Spheres: Attractive Materials for Supercapacitor Electrodes and CO2 Adsorption. Chem. Mater..

[56] Nasini U.B., Bairi V.G., Ramasahayam S.K., Bourdo S.E., Viswanathan T., Shaikh A.U. (2014). Phosphorous and nitrogen dual heteroatom doped mesoporous carbon synthesized via microwave method for supercapacitor application. J. Power Sources.

[57] Edison T.N.J.I., Atchudan R., Sethuraman M.G., Lee Y.R. (2016). Supercapacitor performance of carbon supported Co 3 O 4 nanoparticles synthesized using Terminalia chebula fruit. J. Taiwan Inst. Chem. Eng..

[58] Yu P., Li Y., Zhao X., Wu L., Zhang Q. (2014). Graphene-Wrapped Polyaniline Nanowire Arrays on Nitrogen-Doped Carbon Fabric as Novel Flexible Hybrid Electrode Materials for High-Performance Supercapacitor. Langmuir.

[59] Xu B., Hou S., Zhang F., Cao G., Chu M., Yang Y. (2014). Nitrogen-doped mesoporous carbon derived from biopolymer as electrode material for supercapacitors. J. Electroanal. Chem..

