# Supplementary material for: N-Doped Mesoporous Carbon Prepared from a Polybenzoxazine Precursor for High Performance Supercapacitors
Source: Polymers (Basel). 2021 Jun 22;13(13):2048. doi: 10.3390/polym13132048 (PMC8271977; doi:10.3390/polym13132048)
Supplement: Supplementary file 1 [file polymers-13-02048-s001.zip › polymers-1243340-SI.pdf]

polymers

MDPI

Article

N-Doped Mesoporous Carbon Prepared from a Polybenzoxazine Precursor for High Performance Supercapacitors

Periyasamy Thirukumaran †, Raji Atchudan †, Asrafali Shakila Parveen, Madhappan Santhamoorthy, Vanaraj Ramkumar and Seong-Cheol Kim *

School of Chemical Engineering, Yeungnam University, Gyeongsan 38541, Korea; thiru.kumaran999@gmail.com (P.T.); atchudanr@gmail.com (R.A.); shakilaasraf@gmail.com (A.S.P.); santham83@gmail.com (M.S.); sriram27@gmail.com (V.R.)
* Correspondence: sckim07@ynu.ac.kr
† These authors contributed equally to this work.

Citation: Thirukumarana, P.; Atchudana, R.; Parveena, A.S.; Santhamoorthya, M.; Ramkumara, V.; Kima, S.-C. N-Doped Mesoporous Carbon Prepared by Polybenzoxazine Precursor for High Performance Supercapacitor. *Polymers* **2021**, *13*, 2048. https://doi.org/10.3390/polym13132048

Academic Editor: Michael R. Buchmeiser

Received: 17 May 2021
Accepted: 12 June 2021
Published: 22 June 2021

Publisher's Note: MDPI stays neutral with regard to jurisdictional claims in published maps and institutional affiliations.

Copyright: © 2021 by the authors. Licensee MDPI, Basel, Switzerland. This article is an open access article distributed under the terms and conditions of the Creative Commons Attribution (CC BY) license (http://creativecommons.org/licenses/by/4.0/).

Synthesized of benzoxazines monomers (HPh-Bzo).

In a three necked round bottomed flask equipped with a magnetic stirrer and a reflux condenser, paraformaldehyde (18.0 g, 0.6 m), was taken and 100 mL of DMSO was added to it. It was then allowed to stir while maintaining the temperature at 70°C. phenylethylamine (24.2 g, 0.2 m) was added dropwise to the stirring mixture. In the meantime, a solution containing hydroquinon (11.0 g, 0.1 m) in 20 mL of DMSO was prepared separately. After complete addition of phenylethylamine, the solution of hydroquinon was added dropwise to the reaction mixture. The temperature was then slowly raised to 120°C. The reaction was then continuously stirred for 5h at this temperature. On completion of the reaction time, a transparent pale yellow colored solution was obtained. This solution was then cooled to room temperature and precipitated in 1N NaOH solution. The precipitate thus obtained was washed with distilled H_2O several times, filtered and finally dried in vacuum at 60°C for 12 h to obtain the HPh-Bzo monomer (Scheme S1).

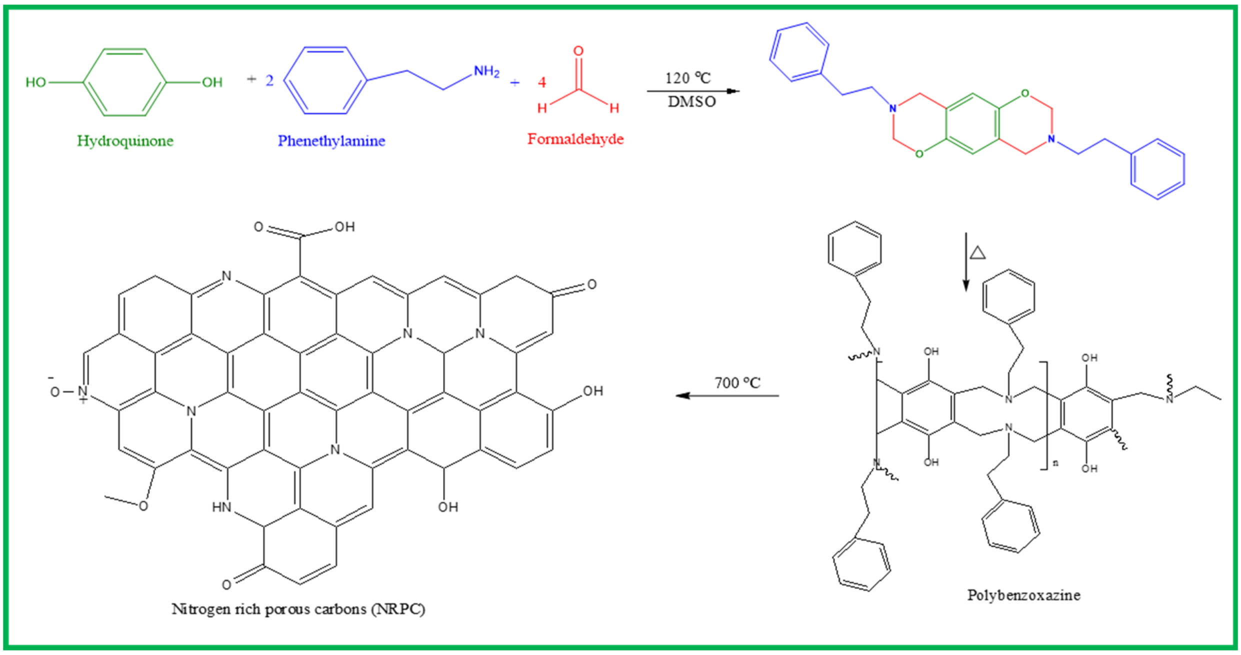

Scheme S1. Synthesized of benzoxazines monomers (HPh-Bzo).

Characterization of the benzoxazine monomer.

The precursor of the polybenzoxazine-based nitrogen-rich carbons was synthesized by the Mannich reaction. Novel porous and high surface area carbons were obtained after carbonization and activation. Fourier transform infrared (FTIR) spectrum of the benzoxazine monomer (HPh-Bzo) was recorded and is shown in Figure S1. The specific adsorption band of at 2848 cm^{-1} and at 2963 cm^{-1} It's conforming to the symmetric and anti-symmetric stretching modes of the CH_2 groups of in between oxazine ring and amine groug, and contribution from the symmetric and anti-symmetric stretching vibration in CH_2 of oxazine ring respectively[1]

There is no brad peak at 3300 cm^{-1} due to all –OH groups are completely react with an amine group to form an oxazine ring. The peak at 1654 cm^{-1}, due to the carbonyl stretching vibration of apigenin moiety. The characteristic absorptions of the oxazine ring structure of HPh-Bzo were seen at1024 and 1228 cm^{-1} attribute to the symmetric and asymmetric stretching modes of C–O–C, respectively, while the peak at 939 cm^{-1} approves the formation of an oxazine ring. Also, the spectrum shows a peak at 1148 cm^{-1} due to the C–N–C symmetric stretching vibrations.The ^{1}H-NMR spectrum shown in Fig. S1 further confirms the structure of HPh-Bzo monomer having oxazine rings has resonance peaks at 4.7 ppm and 3.7, ppm that are assigned to Ar–CH_2–N and O–CH_2–N of the oxazine ring, respectively. The methylene protons (–CH2) connecting the amine group its appeared between 2.5 and 3.5 ppm. Aromatic protons appear between 7.0 and 7.6 ppm.The 13C-NMR spectra also confirm the structures of HPh-Bzo The typical carbon resonance of the oxazine ring is found at 82, and 52ppm for Ar– CH2–N and O–CH2–N, respectively. The methylene carbons (–CH2) connecting the amine group its appeared between e peaks between 110 and 120 ppm. Other aromatic carbons appear in between 120 and 150 ppm. The peak at 40 ppm is due to the solvent peak (DMSO

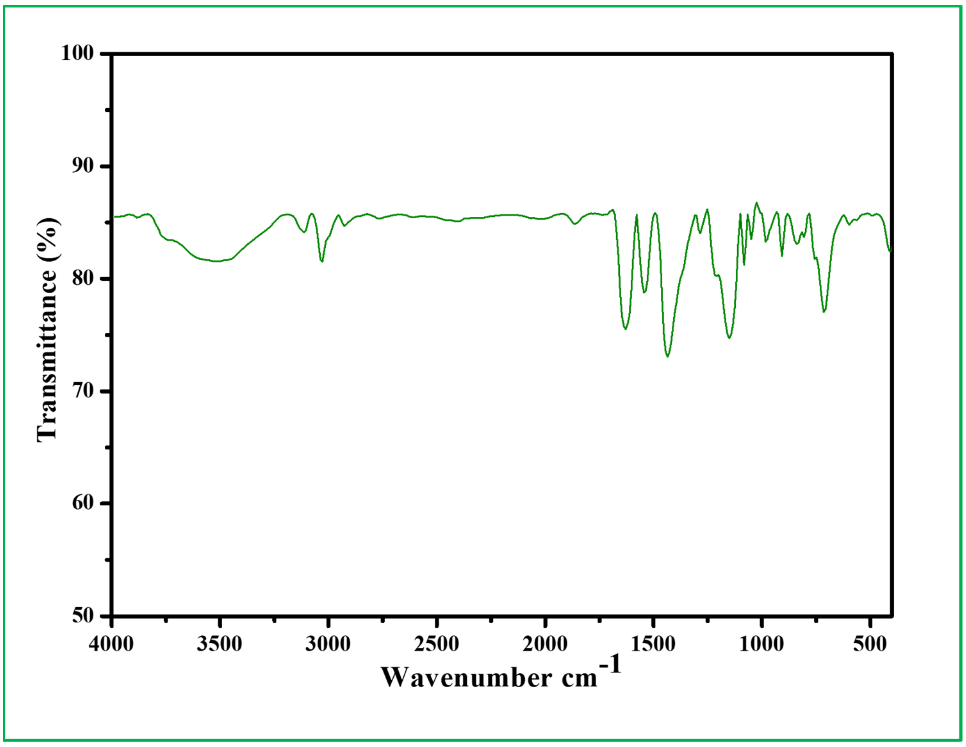

Figure S1. FT-IR spectrum benzoxazines monomers (HPh-Bzo).

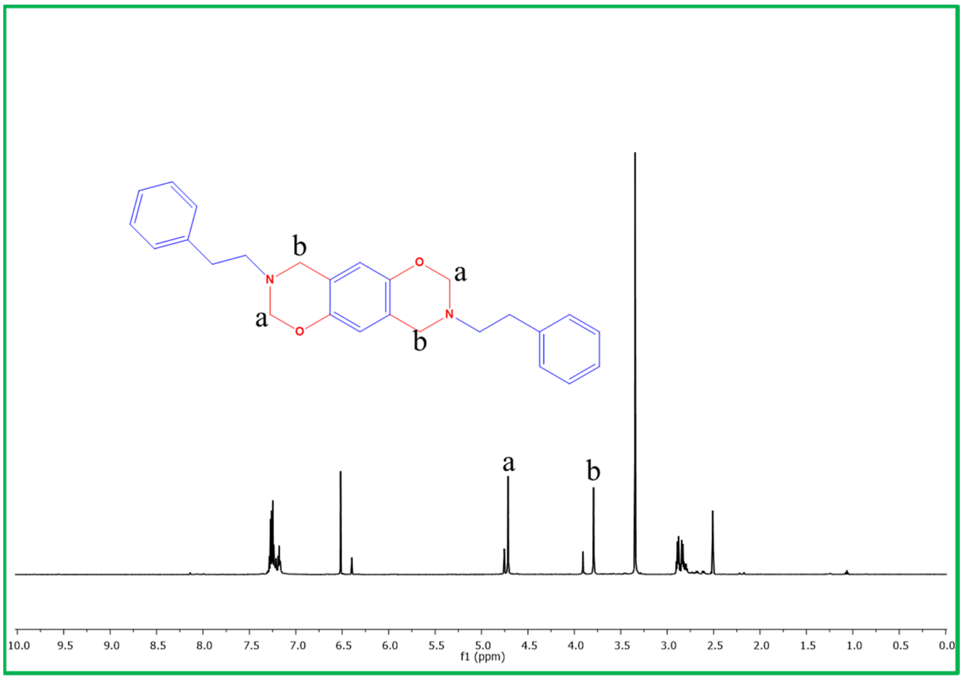

Figure S2. ^{1}H-NMR spectrum benzoxazines monomers (HPh-Bzo).

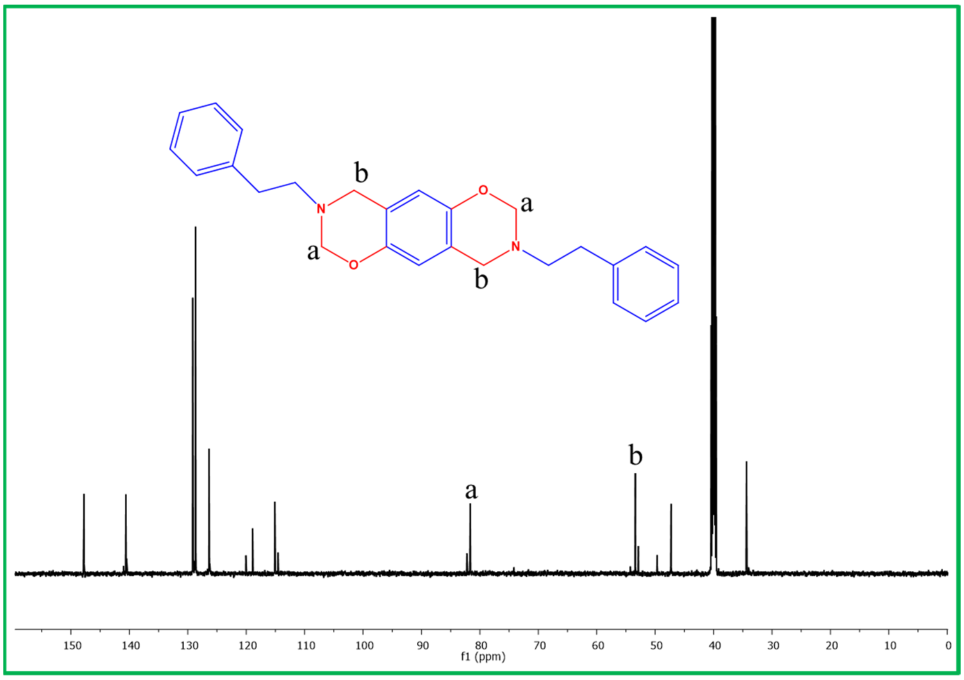

Figure S3. ^{13}C-NMR spectrum benzoxazines monomers (HPh-Bzo).
